# Transgenic substitution with Greater Amberjack *Seriola dumerili* fish insulin 2 in NOD mice reduces beta cell immunogenicity

**DOI:** 10.1038/s41598-019-40768-3

**Published:** 2019-03-21

**Authors:** Kylie S. Foo, Alicja A. Skowronski, Danielle Baum, Rebuma Firdessa-Fite, Sebastian Thams, Linshan Shang, Rémi J. Creusot, Charles A. LeDuc, Dieter Egli, Rudolph L. Leibel

**Affiliations:** 1 Division of Molecular Genetics, Department of Pediatrics and Naomi Berrie Diabetes Center, Columbia University, New York, NY USA; 2 Present Address: Integrated Cardio Metabolic Center, Department of Medicine, Karolinska Institutet, Stockholm, Sweden; 3 Department of Medicine, Columbia Center for Translational Immunology and Naomi Berrie Diabetes Center, Columbia University, New York, NY USA; 4 Departments of Pathology and Cell Biology, Center for Motor Neuron Biology and Disease, Columbia University Medical Center, New York, NY USA; 5 Present Address: Department of Clinical Neuroscience, Karolinska Institutet, Stockholm, Sweden; 6 Present Address: Department of Biochemistry, Molecular Biology, and Biophysics, University of Minnesota, Minneapolis, USA

## Abstract

Type I diabetes (T1D) is caused by immune-mediated destruction of pancreatic beta cells. This process is triggered, in part, by specific (aa 9–23) epitopes of the insulin Β chain. Previously, fish insulins were used clinically in patients allergic to bovine or porcine insulin. Fish and human insulin differ by two amino acids in the critical immunogenic region (aa 9–23) of the B chain. We hypothesized that β cells synthesizing fish insulin would be less immunogenic in a mouse model of T1D. Transgenic NOD mice in which Greater Amberjack fish (*Seriola dumerili*) insulin was substituted for the *insulin 2* gene were generated (mouse *Ins1*^−/−^ mouse *Ins2*^−/−^ fish *Ins2*^+/+^). In these mice, pancreatic islets remained free of autoimmune attack. To determine whether such reduction in immunogenicity is sufficient to protect β cells from autoimmunity upon transplantation, we transplanted fish *Ins2* transgenic (expressing solely *Seriola dumerili Ins2*), NOD, or B16:A-dKO islets under the kidney capsules of 5 weeks old female NOD wildtype mice. The B:Y16A Β chain substitution has been previously shown to be protective of T1D in NOD mice. NOD mice receiving *Seriola dumerili* transgenic islet transplants showed a significant (p = 0.004) prolongation of their euglycemic period (by 6 weeks; up to 18 weeks of age) compared to un-manipulated female NOD (diabetes onset at 12 weeks of age) and those receiving B16:A-dKO islet transplants (diabetes onset at 12 weeks of age). These data support the concept that specific amino acid sequence modifications can reduce insulin immunogenicity. Additionally, our study shows that alteration of a single epitope is not sufficient to halt an ongoing autoimmune response. Which, and how many, T cell epitopes are required and suffice to perpetuate autoimmunity is currently unknown. Such studies may be useful to achieve host tolerance to β cells by inactivating key immunogenic epitopes of stem cell-derived β cells intended for transplantation.

## Introduction

In type I diabetes (T1D), insulin-producing pancreatic β cells are impaired and/or lost through immune-mediated mechanisms. Affected individuals require exogenous insulin to survive. Allogeneic cadaveric islet transplantation can restore euglycemia transiently, but half of all the recipients require exogenous insulin five years post-transplantation^1^.

**Figure 1:**
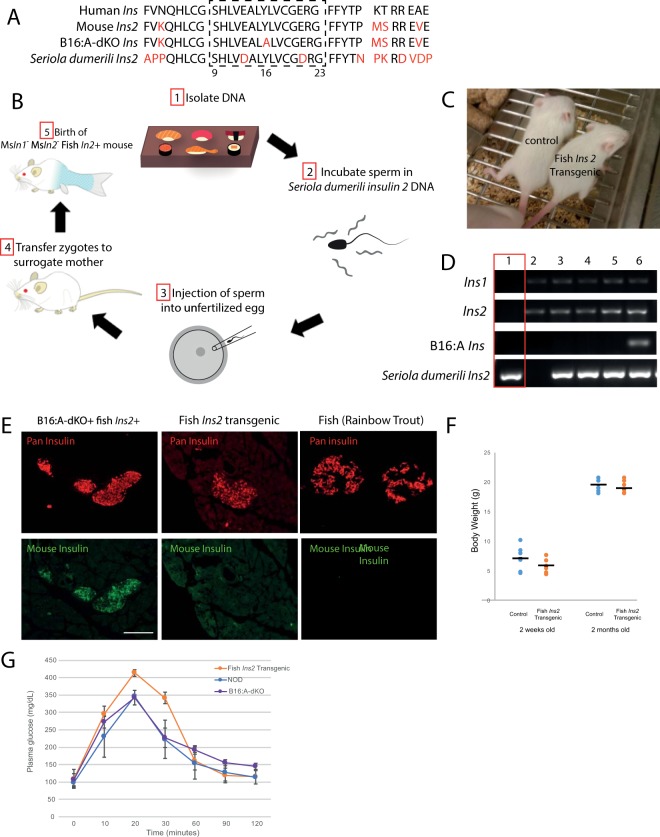
Generation of *Seriola dumerili Ins2* Mouse. (**A**) Sequence comparison of human, mouse *Ins 2*, B16:A-dKO *Ins2*, and *Seriola dumerili* (amberjack) *Ins 2* B chain sequences. Red colored texts indicate difference in amino acid sequence versus human. Dashed box denotes critical region in the B chain 9–23. (**B**) Schematic illustrating the generation of *Seriola dumerili Ins2* transgenic mouse. (**C**) Fish transgenic on right with wildtype control at P14. (**D**) PCR confirmation of *Seriola dumerili* transgenic genotype. Band sizes of specific alleles: mouse *Ins 1* (324 bp), mouse *Ins 2* (198 bp), B16:A *Ins* (318 bp), *Seriola dumerili Ins2* transgene (340 bp). *Seriola dumerili* transgenic (lane 1) does not contain endogenous mouse *Ins 1* or *Ins 2* gene, only fish *Ins2*. (**E**) Pan insulin antibody detects both fish and mouse insulin. Immunohistochemistry of *Seriola dumerili* transgenic pancreata shows expression of fish (top middle panel) but not mouse insulin (bottom middle panel); similar to dissected rainbow trout pancreas (right most panel). Scale bar: 100 um. (**F**) Body weight graph on 2 weeks and 2 months old *Seriola dumerili* transgenic compared to their littermates (n = 6 per group). (**G**) Intraperitoneal glucose tolerance tests on 4-week old NOD, B16:A-dKO, *Seriola dumerili* transgenics (n = 3 in each group; mean ± SEM).

Fish insulin was one of the first vertebrate insulins isolated and sequenced^2,3^. Moreover, fish insulin was used to treat individuals with insulin-dependent diabetes in the early 1940s; particularly in patients who developed neutralizing antibodies against bovine and porcine insulins^4,5^. The Great Amberjack (*Seriola dumerili*), similar to mouse, contains two insulin genes: *Ins 1* and *Ins 2*^6–8^; with the highly conserved *Ins 2* being the closer homologue of the human insulin gene. Fish insulin is functionally active in humans, and shows little or no immunological cross-reactivity with human insulin, partly due to the small differences in its amino acid sequence (Fig. 1A)^9–11^. In a small study, 45 units of tuna fish insulin were administered daily to patients with T1D and was more effective than 100–145 units of bovine insulin given daily in preventing ketoacidosis over an eight day period^12^.

The non-obese diabetic (NOD) mouse develops autoimmune diabetes spontaneously^13^. Early work by Wegmann *et al*. suggested that insulin played an important role as an auto-antigen in the pathogenesis of autoimmune diabetes^14,15^. They showed that in NOD mice, CD4+ T cells participate in early islet infiltrates, and that these insulin-reactive T cells recognized the 9–23aa epitope of the insulin B chain (B:9-23). Eisenbarth and others found that the NOD-specific MHC Class II allele (I-A^g7^; highly homologous to the high risk human HLA-DQ8 MHC Class II allele^15,16^) shows weak binding affinity for insulin self-epitopes, possibly facilitating autoreactive T-cell escape from thymic deletion^16,17^. This faulty binding is due to an incompatibility between the insulin peptide and the MHCII binding register, and has been associated with increased susceptibility to T1D^16^. A single amino acid substitution in the insulin Β chain at position 16 (B:Y16A) in NOD mice null for *Ins1* and *Ins2;* B16:A-dKO (*Ins1*^−/−^ *Ins 2*^−/−^. Ins2*Y16A), prevents the development of autoimmune diabetes^18,19^, supporting the observation that insulin, and the B9–23 epitope in particular, is clearly an important auto-antigen in the modulation of susceptibility to diabetes in mice and humans^20–22^.

The amino acid sequence of the critical region of the insulin 2 B:9–23 is highly conserved across species (Fig. 1A), and there are mouse CD4+ and CD8+ T cell epitopes which react specifically with this segment of insulin gene (Table 1)^23^. We hypothesized that NOD mouse β cells expressing solely *Seriola dumerili Ins2* (mouse *Ins1*^−/−^, mouse *Ins2*^−/−^, fish *Ins2*^+/+^) would be less diabetes-prone by virtue of glutamic acid to aspartic acid substitutions at aa13 and aa21 in *Seriola dumerili Ins2* vs. human insulin in the region of the Β chain essential for immune tolerance to insulin (Fig. 1A). We further postulated that islets isolated from mice expressing solely *Seriola dumerili Ins2* would be better tolerated when transplanted into diabetics-prone female NOD mice. These experiments have implications for strategies to generate clinically transplantable stem cell-derived β cells with reduced immunogenicity through alterations of major epitopes recognized by autoreactive T cells.

**Table 1:** Library of known epitopes on mouse insulin.

Mouse CD4+ T cell epitopes for mouse β-cell insulin auto-antigen	Position	Sequence	AA Differences from Fish *Ins2*
Ins1 and Ins2	A7–21	C**TSI**C**SLYQ**L**E**NYCN	8
Insulin 1	L7–23	**FLPL**L**AL**L**ALWEPKPT**Q	14
	L20-B11	**KPTQAFVK**QHLCG**P**HL	9
	B9–23	**P**HLV**E**ALYLVCG**E**RG	3
	C15–30	**SPGDLQTLAL**E**V**A**RQK**	14
	C21-A5	**TLALEVAR**QKRGIV**D**Q	9
Insulin 2	L14-B6	L**FLWESHPTQAFVK**QHL	13
	L20-B11	**HPTQAFVK**QHLCGSHL	8
	B2–17	**VK**QHLCGSHLV**E**ALYL	3
	B9–23	SHLV**E**ALYLVCG**E**RG	2
	B24-C1	FFY**T**P**MSRRE**	6
	C15–32	**GP**G**AGDLQTLALEVA**Q**QK**	16
**Mouse CD8+ T cell epitopes for mouse β -cell insulin autoantigen**	**Position**	**Sequence**	**AA Differences from Fish** ***Ins2***
Insulin 1/2	B15–23	LYLVCG**E**RG	1
Insulin 2	B25-C2	FY**T**P**MSRREV**	7

A list of known mouse CD4+ and CD8+ T cell epitopes for mouse insulin auto-antigen. Bold lettering indicates the sequence differences between mouse insulin 1, 2, and *Seriola dumerili Ins2*.

## Results

### Mice exclusively expressing *Seriola dumerili Ins2* are viable

**Figure 2:**
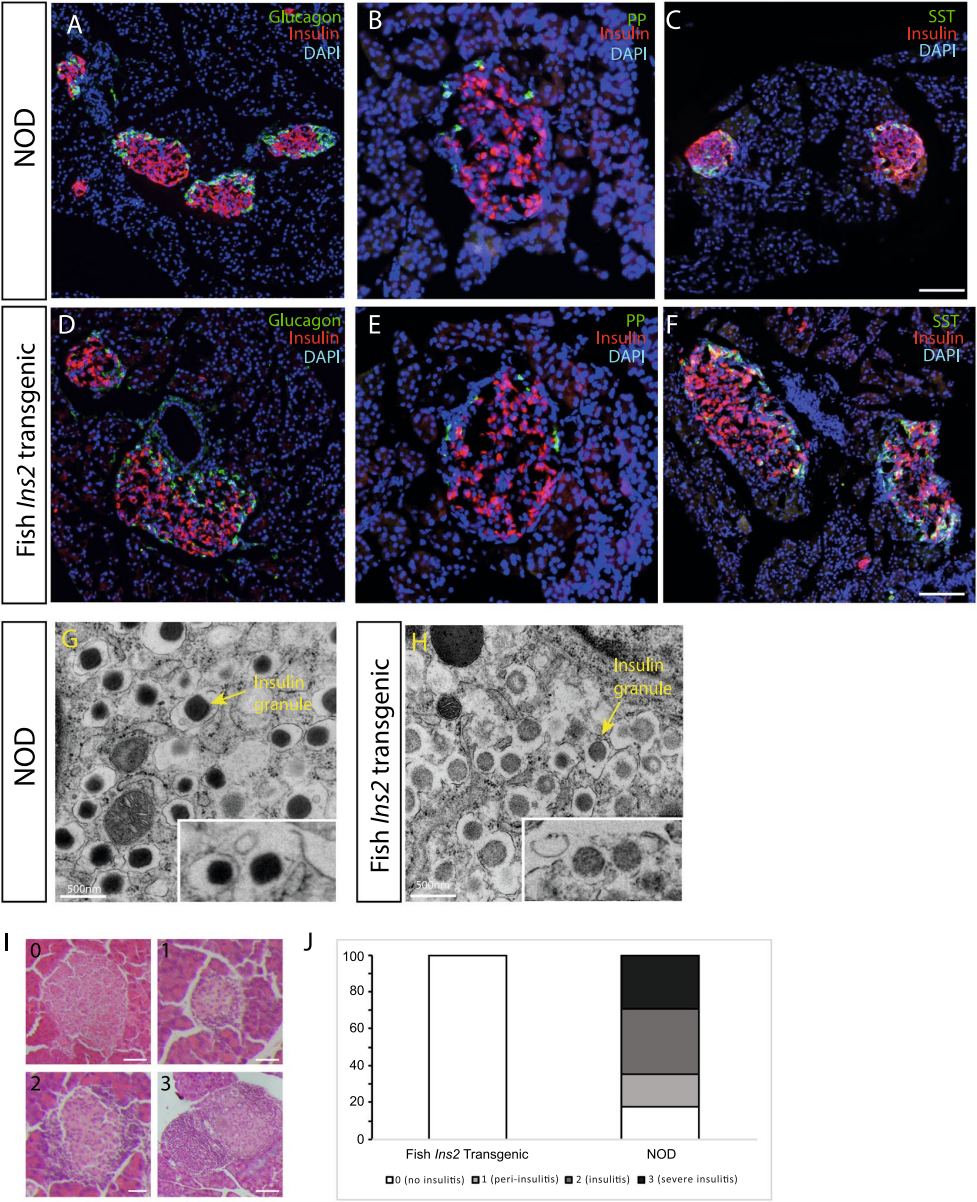
Histologic comparison of wild type and *Seriola dumerili* mouse pancreata. *Seriola dumerili Ins2* mice (bottom) have normal islet morphology and cyto-architecture compared to littermates with endogenous mouse *Ins 1* and *Ins 2* (top); insulin (red **A**–**F**), glucagon (green; **A**,**D**), pancreatic polypeptide (green; **B**,**E**), and somatostatin (green; **C**,**F**). **G**, **H**: Beta cell ultra-structure of NOD and *Seriola dumerili Ins2* transgenic. Both NOD (**G**) and *Seriola dumerili Ins 2* transgenic (**H**) islets contain insulin granules (yellow arrow), though *Seriola dumerili Ins2* transgenic insulin granules are lighter in staining intensity compared to the NOD (n = 4; zoom in; inset). (**I**,**J**) Insulitis scoring pancreata from 12–15 weeks old NOD and *Seriola dumerili Ins2* transgenics (n = 4 per group). Scale bar: 100 um.

Mice expressing *Seriola dumerili Ins2* were generated by microinjection of *Seriola dumerili Ins2* transcripts incubated with B16:A-dKO mouse sperm into NOD oocytes (Fig. 1B). The F1 generation yielded 6 live births with offspring segregating for mouse insulins and, potentially, for B16:A and/or fish *Ins2*. One of the seven progeny expressed the *Seriola dumerili Ins2* transgene. This founder mouse was crossed with NOD mice (Jax cat no. 001976) and their fish *Ins2*-expressing progeny were serially inter-crossed to wildtype NOD to breed out the B16:A, mouse *Ins1* and *Ins2* alleles until only the *Seriola dumerili Ins2* transgene remained (Fig. 1C–E). *Seriola dumerili* transgenic mice were viable and fertile. PCR confirmed that these mice expressed *Seriola dumerili Ins2* exclusively (Fig. 1D, red box). Immunohistochemistry also showed that *Seriola dumerili* transgenic mice expressed fish *Ins2* (Fig. 1E), but not native mouse insulin (Fig. 1E). A polyclonal pan insulin antibody (Dako A0564) reactive against mouse, and zebrafish was used to detect the presence of *Seriola dumerili* insulin. The fish insulin genotype did not affect overall islet morphology or the locations of β-cells, α-cells, δ-cells, and PP cells (Fig. 2A–F).

At 2 weeks of age, *Seriola dumerili* transgenic pups trended to a lower body mass than control NOD (n = 6; student t test, not significant, Fig. 1F). *Seriola dumerili Ins2* is functionally active, since insulin-deficient mice showed early neonatal lethality within 48 hours after birth^22^. Capillary glucose concentrations at 2 weeks of age did not differ between *Seriola dumerili Ins2* transgenic, and NOD mice. All were euglycemic; none were glycosuric. At 4 weeks of age, an intraperitoneal glucose tolerance test was normal in animals (n = 3 per group, Fig. 1G); plasma glucose concentrations peaked at 20 minutes and returned to basal levels at 90 minutes. At the 15–30 min time point, there was a non-significant trend to higher plasma glucose levels in *Seriola dumerili Ins2* transgenic compared to both NOD and B16:A-dKO (one-way ANOVA). These results suggest that fish *Ins2* was sufficient to meet the metabolic demands of a developing mouse up to one month of age.

By electron microscopy, islets of fish *Ins2* transgenic mice contained densely packed secretory granules (Fig. 2G). These were ~ 300 nm in diameter and had electron-dense cores. Crystalline forms were prominent in the cores of the *Seriola dumerili* transgenic granules, characteristic of secretory granules of normal β cells (Fig. 2H). Overall, compared to the control islets (NOD), the electron-dense cores of fish *Ins2* transgenic islets were lightly stained as opposed to the darker dense cores in the NOD (n = 3 per group).

Even at 32 weeks of age, *Seriola dumerili* transgenic mice showed no evidence of islet infiltration with immunocytes (Fig. 2I,J). By comparison, most female NOD mice (bred in house; incidence of diabetes in our colony is 85% for females by age of 30 weeks when housed in our pathogen free barrier facility) showed histological evidence of insulitis (Fig. 2J; 12–32 weeks of age; n = 4 per group).

At 4 weeks of age, fish *Ins2* transgenic mice were euglycemic. However, starting at 5 weeks of age, all male and female *Seriola dumerili Ins2* transgenic mice became diabetic (plasma glucose concentrations consistently over 600 mg/dL) and required exogenous insulin administration for metabolic support.

The development of hyperglycemia was not due to loss of *Seriola dumerili* insulin- secreting β cells by autoimmunity. The number of islets were similar among the genotypes; the average number of islets was roughly 200 islets per mouse (n = 15 NOD; n = 10 B16:A-dKO; n = 6 *Seriola dumerili Ins2* transgenics). Even at 32 weeks of age, there were no signs of inflammatory cell infiltrates in the fish transgenic islets. In contrast, the islets of NODs were heavily infiltrated (Fig. 2J). After 1 month of age, male and female *Seriola dumerili Ins2* transgenic mice that were supported metabolically with subcutaneous exogenous insulin pellets showed no evidence of insulitis, suggesting that the deterioration of their glucose homeostasis was most likely due to insufficient insulin transgene expression.

### Lymphocyte infiltration is delayed in *Seriola dumerili* transgenic islet grafts

**Figure 3:**
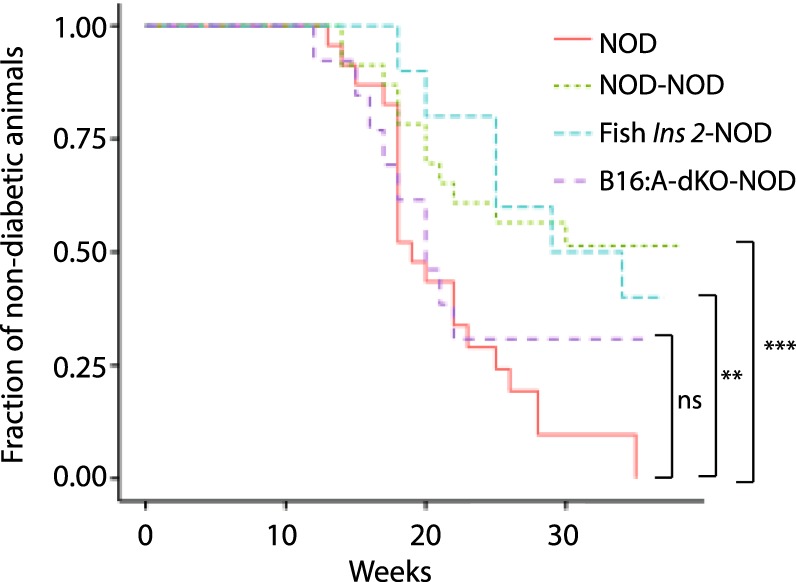
Cumulative Incidence of diabetes in NOD transplanted with NOD, B16:A-dKO or *Seriola dumerili Ins 2* transgenic islets. Islets were isolated from NOD, B16:A-dKO, or *Seriola dumerili Ins2* transgenic mice and transplanted under the kidney capsules of 5-week old NOD female mice before the onset of diabetes. 100 islets from each group were transplanted. Shown are Kaplan-Meier survival curves of treated, wildtype NOD, percentage of diabetes-free mice. NOD receiving *Seriola dumerili* islets (n = 10; in turquoise) had a longer diabetes-free period (up to 24 weeks of age) compared to NOD without any transplanted islets (n = 20 in red; ctrl); ^***^P = 0.004; log-rank test. Those NOD mice receiving NOD islets (n = 23; in green) also significantly increased their diabetes-free period; ^**^P = 0.001; log-rank test. Mice receiving B16:A-dKO islets (n = 16; in purple) did not show prolongation of their diabetes-free period *P* = 0.312; log-rank test. In our vivarium, 50% of female NOD develop diabetes by 18wks of age, and by 26 weeks of age, ~80% are diabetic (in red; ctrl).

**Figure 4:**
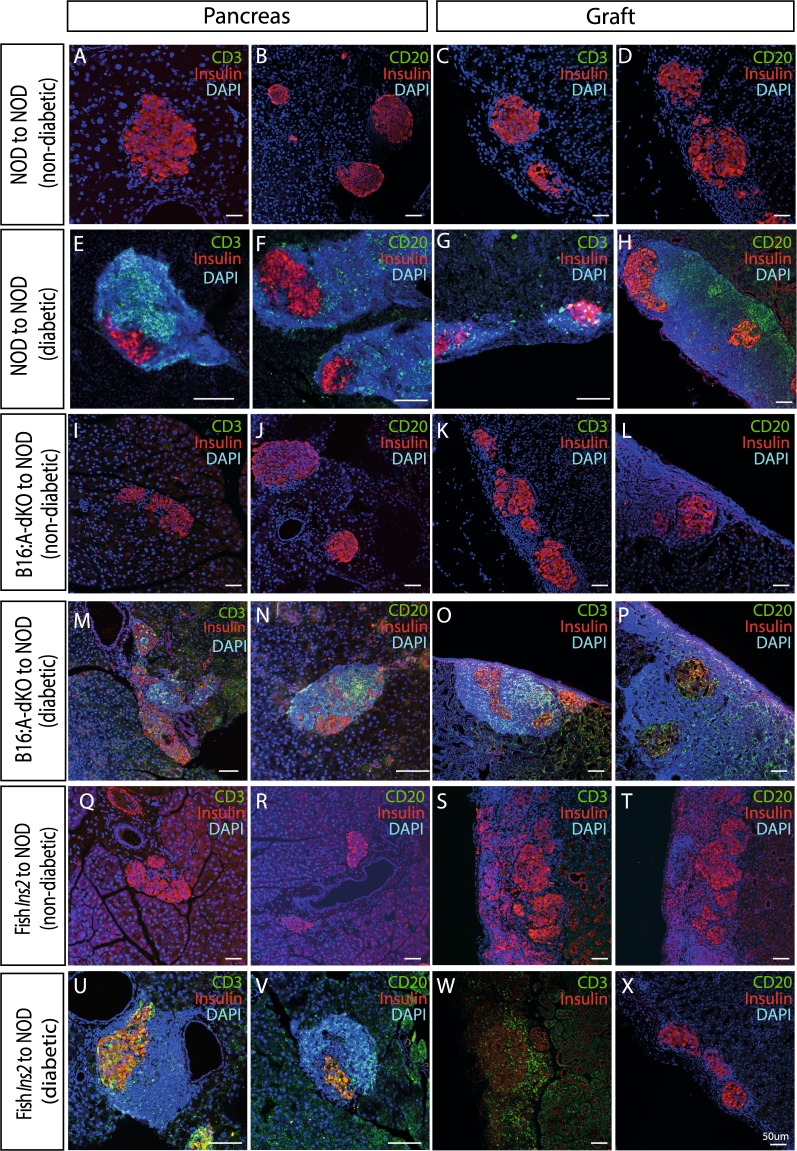
Immunohistochemistry of immune cells in pancreas and under kidney capsules. Top panel A-H are NOD mice that received NOD islets under their kidney capsules and remained normoglycemic (**A**–**D**) or became diabetic (**E**–**H**); I-P are NOD mice that received B16:A-dKO islets: some animals remained normoglycemic (**I**–**L**), others became diabetic (**M**–**P**); (**Q**–**X**) are NOD mice that received *Seriola dumerili Ins2* transgenic islets: (**Q–T**) remained normoglycemic, while **U**–**X** are from recipients which became diabetic. Both CD3 and CD20 are present in the pancreata and islet grafts of the NOD mice that received NOD islets (**E**–**H**), and B16:A-dKO islets (**M**–**P**) that became diabetic. A fraction of the NOD receiving *Seriola dumerili Ins2* transgenic islets became diabetic, and both CD3 and CD20 cells are present in pancreas (**U**,**V**), and CD3 can be seen surrounding the *Seriola dumerili Ins2* transgenic islets under the kidney capsule (**W**), but CD20 cells are undetected in islets under the kidney capsule (**X**). Scale bar: 50um; n = 6 per group.

To further assess the immunogenicity of fish *Ins2* transgene, we transplanted islets isolated from *Seriola dumerili Ins2* transgenic under the kidney capsules of healthy 5-week old female NOD mice. We investigated whether transplanted *Seriola dumerili Ins2* transgenic islets are less immunogenic compared to immune cells triggering NOD islets. These healthy 5 weeks old female NOD mice received 100 islets from: 1. Female NOD (n = 23 recipients); 2. B16:A-dKO transgenic (n = 16); or 3. *Seriola dumerili Ins2* transgenic (n = 10). Capillary glucose concentrations were monitored weekly at 10–11 AM in *ad lib* fed animals starting at 10 weeks of age. Transplantation of NOD (^***^P = 0.001, log-rank test; Fig. 3) or *Seriola dumerili Ins2* transgenic islets (^**^P = 0.004, log-rank test; Fig. 3) reduced diabetes incidence in NOD recipient mice. There was no difference in the incidence of diabetes between those receiving B16:A-dKO islets and NOD not receiving exogenous islet transplants (p = 0.312 log-rank test; Fig. 3). Transplanted NOD islets and B16:A-dKO islets were extensively infiltrated with T and B cells after onset of diabetes (Fig. 4M–P) from age of 12 weeks onwards (7 weeks post transplantation). The NOD recipients that remained diabetes-free showed absence of pancreatic islet graft CD3 and CD20 cell infiltrates, regardless of donor islet cellular phenotypes: NOD, B16:A-dKO, or *Seriola dumerili Ins2* transgenic (Fig. 4A–D, I–L, Q–T). After onset of diabetes, the pancreata of the recipient NOD mice showed prominent infiltrates of CD3, and CD20 cells irrespective of transplanted donor islet phenotypes (Fig. 4E,F,M,N,U,V). A portion of NOD mice transplanted with *Seriola dumerili Ins2* transgenic islets ultimately became diabetic (50% by end of study at 38 weeks of age) and their pancreata and grafts were analyzed for CD3 (T cell marker), and CD20 (B cell marker) cells. These cells were present in the pancreata of the recipient NOD mice (Fig. 4U,V). The *Seriola dumerili Ins2* transgenic islet transplants in NOD recipients that later became diabetic were infiltrated with CD3 but not CD20 positive cells (n = 4; Fig. 4W,X; Table 2). Although islets isolated from *Seriola dumerili Ins2* transgenic did not completely prevent the onset of diabetes, they did prolong the diabetes free period by up to 6 weeks, and reduced the incidence of diabetes in recipient NOD mice.

**Table 2:** Status of immune infiltration in pancreas and in graft post islet transplantation into NOD mouse.

Islet Donor-Recipient	Diabetes status	Pancreas	Graft
NOD-NOD	Non-diabetic	+	−
	Diabetic	+	+
B16:A-dKO -NOD	Non-diabetic	+	−
	Diabetic	+	+
*Seriola dumerili Ins2* - NOD	Non-diabetic	+	−
	Diabetic	+	**+/− only T cells**

A table summarizing immune infiltration responses in pancreas and grafts of NOD recipients post islet transplantation.

### *Seriola dumerili Ins2* does not activate responsiveness of CD4+ T cells

**Figure 5:**
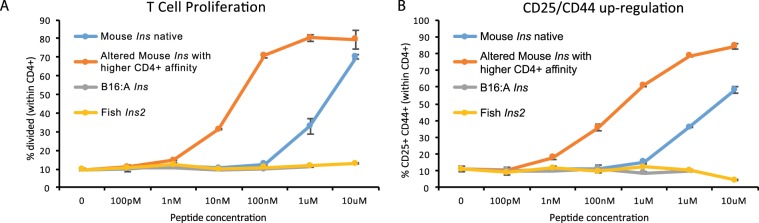
*Seriola dumerili Ins2* does not activate responsiveness of CD4+ T cells. A series of insulin B:9–23 peptide variants: 1) native mouse Ins2B:9–23 (blue); 2) R22E mutant peptide of mouse Ins2B:9–23 recognized with higher affinity (positive control); 3) Y16A mutant peptide from B16:A insulin (negative control), and *Seriola dumerili* Ins2B:9–23 peptide were examined. *In vitro* T cell proliferation assay (**A**) showed that both native and R22E-modified peptides trigger a proliferative response, while peptides from B16:A-dKO and *Seriola dumerili* insulin failed to elicit any T cell proliferative response even at the highest peptide concentration of 10 μM. (**B**) Similarly, there was strong CD4 up-regulation of activation markers with both native mouse insulin and mimotope peptides, whereas peptides from B16:A-dKO insulin and *Seriola dumerili Ins2* did not activate the CD4+ T cell clone.

We investigated the interactions among different insulin epitopes and T cells. We performed *in vitro* T cell proliferation and activation assays to examine the responsiveness of CD4+ T cells from BDC12-4.1.TCRαKO transgenic mice^24^ (which are reactive to InsB:9–23) to a series of truncated insulin B:9–23 peptide variants: 1) native mouse insulin InsB:9–23; 2) R22E mutant peptide (p8E mimotope)^25^ of mouse Ins2B:9–23 recognized with higher affinity (positive control); 3) Y16A mutant peptide from B16:A insulin (negative control); and 4) *Seriola dumerili* Ins2B:9–23 peptide with the same amino acid sequence as in the *Seriola dumerili* transgenic islets transplanted into NOD mice (Fig. 1A). There was extensive T cell proliferation with both native peptide and R22E mimotope, while peptides from both B:Y16A *Seriola dumerili* insulins failed to induce any proliferation (Fig. 5A). Similarly, endogenous mouse insulin elicited strong T cell stimulation, as reflected by CD25 and CD44 up-regulation, while B16:A insulin and *Seriola dumerili Ins2* did not trigger T cell activation (Fig. 5B). These data are indicative of a lack of immunogenicity of *Seriola dumerili Ins2* for this T cell clone.

## Discussion

In this study, we show that mouse β cells expressing exclusively *Seriola dumerili Ins2*, are functional, and can delay onset of autoimmunity in female NOD. Expression of *Seriola dumerili Ins2* alone supported metabolic sufficiency in mice up to 4 weeks of age. However, these animals became diabetic shortly thereafter. To determine whether substitution of fish *Ins2* was sufficient to protect β cells in the presence of an ongoing immune response, we grafted female NOD mice with *Seriola dumerili Ins2-* expressing islets prior to diabetes onset. As female NOD mice age, endogenous β cells of these mice are destroyed by infiltrates of both T and B cells^26,27^. Engraftment with islets expressing fish *Ins2* gene was associated with a delay of diabetes onset at 6 weeks post transplantation; however, these grafts nonetheless showed T cell infiltrates.

The deterioration of glucose homeostasis of *Seriola dumerili Ins2* transgenic mice after 4 weeks of age was not due to autoimmunity, but likely the result of lower bioactivity of fish *Ins2* and/or reduced expression of the transgene. Upon provision of supplemental exogenous insulin, these animals were euglycemic and showed no histological evidence of insulitis. In autoimmune T1D- prone NOD mice, *Ins1* and *Ins2* genes are located on different chromosomes with different thymic tolerances^28^. *Ins2* is expressed in both pancreatic islets and thymus, while the *Ins1* gene is expressed only within the islets^29,30^. It has been shown that knockout of *Ins2* in NOD mice accelerates diabetes while knockout of *Ins1* eliminates insulitis and diabetes in female mice^29,30^. Similar to the NOD *Ins1* knockout, our *Seriola dumerili Ins2* transgenic mice did not display insulitis in the context of the onset of diabetes after 4 weeks of age. The effects of insulin gene dosage probably played a role in the onset of diabetes our *Seriola dumerili Ins2* transgenic animals. Mice with one copy of *Ins1* gene (null for *Ins2*) when fed a high fat diet, show deterioration of glucose homeostasis^31^. It is plausible that heterozygosity for functional *Ins 2* would have a similar effect. The lack of immune cell infiltrations in our single founder *Seriola dumerili Ins2* transgenics colony cannot be unequivocally attributed to the modification of the insulin epitopes. The presumed lower expression level of the fish *Ins 2* transgene could potentially contribute to the lack of infiltration in the *Seriola dumerili Ins2* transgenics, since thymic expression of transgene driven by the rat insulin promoter can result in transgene-specific systemic tolerance^32^.

Our results are consistent with the studies by others, demonstrating that a B:Y16A substitution can mitigate autoimmunity and diabetes in NOD mice^18,19^. We attempted to quantify circulating fish insulin levels, and secretion levels by freshly isolated *Seriola dumerili Ins2* transgenic islets. However, there was only one fish insulin ELISA kit commercially available when this study was conducted, and it failed to detect secreted *Seriola dumerili Ins2*, or insulin in a plasma sample of rainbow trout.

The 100 transplanted *Seriola dumerili Ins2* transgenic islets could not ultimately sustain glucose homeostasis of the NOD recipients. This outcome in itself is not surprising given the fact that these transplants represented only 5–10% of normal mouse islet mass^33^. However, these islets did apparently mitigate disease progression via mechanisms possibly similar to administration of B:9–23 peptide analogs with alanine substitutions at residues 16 and 19 in NOD mice^34^. Both *Alleva et al*. and our study utilize amino acid substitutions in a critical region of the insulin B chain, and resulted in delayed destruction of these transplanted islets. This delay may be partially due to the failure of particular mouse T cell clones to detect fish insulin as suggested by our *in vitro* T cell assay. Therefore, while endogenous islets expressing an altered insulin B chain are protected from immune assault, transplants derived from these islets are not fully protected in the presence of an ongoing autoimmune response that was initiated by the endogenous pancreas containing β cells expressing mouse insulin. Once autoimmunity is triggered by insulin-specific T cells, T cells directed at other antigens may contribute to β cell destruction^35^. Questions regarding the cellular and molecular events that trigger and propagate epitope spreading remain. However, this study supports the extensive literature implicating the insulin B:9–23 peptide as a primary auto-antigen in autoimmune diabetes^15–18,36–38^. Allelic variation of the MHC II molecule, by influencing insulin B:9–23 binding, confers susceptibility to T1D in both NOD mice and humans^39–41^. The B:9–23 amino acid sequences of mouse *Ins*2 and human *INS* are identical^30^. The effect of *Seriola dumerili* fish B:E13D appears comparable to that of B16:A-dKO mice in reducing immune assault on endogenous islet β cells in NOD mice^17^.

Given the central role of insulin as a self-antigen and the 9–23 amino acid region of the insulin B chain is an immunodominant T-cell target antigen that plays a critical role in the disease process, immunotherapies based on B:9–23 peptide analogs have been studied in mice and humans for their therapeutic potential^33,42,43^. A series of B:9–23 peptide analogs with alanine substitutions notably at aa 16 and 19, and altered peptide ligands capable of inhibiting B:9–23 induced proliferative responses, have been identified in mice^34^.

Subcutaneous administrations of NBI-6024 (a peptide ligand with alanine substitutions at B: residues 16 and 19) to NOD mice before or after onset of diabetes delayed the onset by up to 20 weeks and reduced the incidence of diabetes^33^. These altered peptide ligands are peptide analogs of immune-dominant auto-antigenic epitopes, which have the ability to competitively inhibit pathogenic autoreactive T cell clones from recognizing native peptide epitopes^44–46^. Based on these NOD mouse studies, several clinical trials have been conducted to assess the efficacy of modified insulin peptides in the prevention of T1D^41^. Preliminary results from three early clinical studies showed safety and tolerance of NBI-6024^43,44^. However, a randomized, placebo-controlled, dose-response phase 2 clinical trial with this peptide failed to demonstrate any clinical benefit^42^.

The experiments reported here suggest that modification of the insulin gene in transplanted β cells might improve their survival in recipients with extant T1D. However, our experiments also show that while the congenital substitution of the B:9-23 insulin epitope alone is sufficient to prevent autoimmunity, it is not sufficient to fully suppress assault in NOD animals congenitally sensitized to insulin. Additional modifications to eliminate major T cell epitopes expressed in β cells - without altering function of β cell proteins -  may also be required to enhance β cells viability in individuals with extant T1D. To enable this capability, a comprehensive characterization of β cells T cell epitopes will be important^47,48^.

## Methods

### Animals

The generation of NOD mice with *Ins1* and *Ins2* knockouts, and knock-in of a sequence-modified humanized insulin (B chain B:Y16A) mice has been previously described^11,24,25^. NOD/ShiLtJ (NOD) mice were obtained from Jackson Laboratory. *Ins1*-, *Ins2-* null mice segregating for Ins2*Y16A (B:Y16A), NOD.Cg-Tg (Ins2*Y16A)1EII Ins1^tmJja^ Ins2^tm1Jja^/GseJ (B16:A-dKO)^17^ were also purchased from Jackson Laboratory (cat no. 005525). The generation of BDC12–4.1.TCRαKO mice was previously described^24^. All mice were fed chow *ad libitum* (Purina PicoLab 5058). Mice were housed at an ambient temperature of 22–24 °C with a 12-hour dark and light cycle (lights on at 7am) in a pathogen-free barrier facility.

The Great Amberjack fish (*Seriola dumerili*) contains 2 insulin genes (*Seriola dumerili Ins1* and *Seriola dumerili Ins2*). We used *Seriola dumerili Ins2* DNA for this study. Fish Insulin DNA was isolated from fresh *Seriola dumerili* obtained at a local sushi restaurant (Fig. 1B). *Seriola dumerili* is also marketed as yellowfin tuna at some sushi restaurants. The *Seriola dumerili Ins2* genomic DNA was amplified using PCR primers5′‐ctatcgatagttgcagtagttctgcaggtcg‐3′ and 5′‐ctatcgatcagttgcagtagttctgcagg ‐3′ on Aug 4, 2009, and cloned into a TOPO TA cloning vector and sequenced for confirmation. The insulin gene was subsequently cloned using ClaI (NEB) digestion into a ClaI digested plasmid containing the rat Ins2 promoter (PMID:15129273) and sequenced using Sanger sequencing. Mouse sperm from B16:A-dKO mice were incubated with a linearized plasmid at 1ng/ml  *Seriola dumerili* DNA and rat *Ins2* promoter in 10 mM NaOH, and subsequently neutralized with an equal volume of 10 mM HCl as described^49,50^. Sperm heads were then microinjected into donor eggs from NOD genotype (Fig. 1B). Embryos at (2–4 cell) stage were then transferred to Day 0.5 pseudo-pregnant CD-1 surrogate mothers (Crl: CD1; obtained from Charles River). Mice segregating for the fish *Ins2* transgene were crossed to wildtype NOD mice and progeny selected by genotyping to generate NOD mice expressing only fish *Ins2*. The *Seriola dumerili* transgenic colony was established from a single founder.

Only female mice were used as islet recipients in the transplantation experiments. The incidence of diabetes in our NOD colony (n = 60) is 85% by 28 weeks of age for females when kept under conventional pathogen-free conditions (Fig. 4). Fresh whole rainbow trout fish was purchased from local supermarket, and the Brockmann body (an endocrine organ which secretes insulin in teleost fish) was rapidly dissected and fixed with 4% paraformaldehyde for immunohistochemistry. All experiments were approved by the Columbia University Institutional Animal Care and Use Committee (IACUC), and all experiments were performed in accordance to relevant guidelines and regulations.

### Assessment of diabetes, insulitis

The clinical onset of diabetes was determined by the presence of glucose in the urine and elevated capillary blood glucose concentrations. Urine test strips were used to monitor glycosuria, and capillary tail blood glucose levels were measured with a FreeStyle blood glucose monitoring system (Abbott Laboratories, Chicago). *Ad-lib* fed mice were considered diabetic after 2 consecutive blood glucose values greater than 250 mg/dL obtained in the morning around 10am. For glucose tolerance testing, mice were fasted for 6 hours, and glucose was administered intraperitoneally (1 mg/g body weight); capillary glucose from tail vein was measured before injection, and at 10, 20, 30, 60, 90, and 120 mins post administration.

To score insulitis, pancreata were fixed in 4% formaldehyde solution, dehydrated, and embedded into paraffin; 5 μm sections were stained with H&E and scored for severity of insulitis.

### Immunohistochemistry

Pancreata and islet grafts transplanted under kidney capsules were fixed in 4% PFA overnight and embedded in paraffin. Paraffin-embedded tissue sections were stained with H&E, and sections from islet grafts were also stained with various antibodies. Primary antibodies: goat anti-glucagon (A056501; DAKO), polyclonal guinea pig anti-insulin (A0564; DAKO), rabbit anti-insulin (4590; Cell Signaling), goat anti-pancreatic polypeptide (NB100–1793; Novus Biological), rabbit anti-somatostatin (A0566, DAKO), rabbit anti-CD3 (Abcam), goat anti-CD20 (Santa Cruz). Secondary antibodies against: guinea pig (Alexa 594), goat (Alexa 488), and rabbit (Alexa 488) all from Life Technologies. Polyclonal pan insulin antibody detected both fish and mouse insulin (Fig. 1E). To evaluate cellular infiltrations, islets throughout the entire pancreas from head to tail from an individual mouse were randomly selected. H&E staining was performed on ten randomly selected sections to assess insulitis.

### Transmission electron microscopy

Pancreata were sectioned into 1–2 mm^2^ slices and fixed overnight with 2.5% glutaraldehyde in 0.1 M Sorenson’s buffer (pH 7.2), for 1 hour in preparation of Epon-embedded tissue blocks. Sections of 70 nm thickness were placed on copper grids and imaged. Processing and imaging of the sample was performed by Diagnostic Service, Department of Pathology and Cell Biology, Columbia University. Insulin granules were defined as electron-dense granular structures at magnification of x 7500.

### Islet isolation and transplantation

Pancreatic islets were isolated by injection of collagenase buffer (type V; Sigma-Aldrich) into the common bile duct. The pancreas was removed and incubated at 37 °C for 15 mins in collagenase buffer. The digested pancreas was washed and density gradient centrifugation was performed using Histopaque-1119 (Sigma-Aldrich) to isolate islets. The islets were handpicked under a microscope, and cultured for 3 hours at 37 °C in RPMI 1640 with 10% FBS in a 95% air 5% CO_2_ humidified atmosphere prior to transplantation. Total islet numbers were determined by manual counting. Batches of 100 islets were transplanted beneath the kidney capsules of 5 weeks old female NOD mice.

### *In vitro* CD4+ T-cell stimulation assay

BDC12-4.1.TCRαKO mice were used as donors of T cell receptor transgenic T cells reactive to the Ins2B:9–23 peptide. Splenocytes from these mice were treated with red blood cell lysis buffer and labeled with Violet Cell Proliferation Dye (eBioscience), then plated at 0.2 × 106 cells per well (U-bottom 96-well plates) in complete RPMI medium containing 10%FBS, 50 IU/ml penicillin, 50 μg/ml streptomycin, 2 mM L-glutamine, 0.1 mM non-essential amino acids, 1 mM sodium pyruvate and 0.05 mM of 2-Mercaptoethanol. Ins2B:9–23 (p8E) amino acid sequence: SHLVEALYLVAGEEG. Peptides were added at a final concentration ranging from 100 pM to 10 μM (10-fold dilutions). After 3 days of culture in a humidified CO2 incubator at 37 °C, the cells were harvested and stained with antibodies against mouse CD4, CD25, and CD44 (Biolegend) and analyzed on BD Fortessa for CD4+ T cell activation and proliferation.

### Statistical analyses

Statistical analysis included one-way ANOVA test to test differences due to genotype at the insulin locus, and performed post hoc pairwise comparison using Student’s t-test. Survival curves were analyzed with the log-rank test. Statistical tests were conducted using PRISM software (GraphPad). Statistical significance was denoted as: *P < 0.05, **P < 0.01 and ***P < 0.001.
